# Kinematic Assessment Utilizing Xsens Gait Motion Analysis in Upper Cross Syndrome: A Case Report

**DOI:** 10.7759/cureus.60485

**Published:** 2024-05-17

**Authors:** Vaishnavi R Waghe, Shrushti Jachak, Raghumahanti Raghuveer, H V Sharath

**Affiliations:** 1 Department of Neurophysiotherapy, Ravi Nair Physiotherapy College, Datta Meghe Institute of Higher Education and Research, Wardha, IND; 2 Department of Pediatrics, Ravi Nair Physiotherapy College, Datta Meghe Institute of Higher Education and Research, Wardha, IND

**Keywords:** posture, rehabilitation, physiotherapy, upper cross syndrome, kinetic analysis

## Abstract

Upper crossed syndrome (UCS) characterizes a prevalent postural dysfunction involving dysfunctional tone in the musculature of the shoulder girdle and cervicothoracic region. The discordant balance among the sternocleidomastoid, pectoralis major, levator scapulae, and upper trapezius musculature potentially precipitated cervical discomfort, thereby hindering routine activities and fostering the progression of UCS. Clinical scales are routinely utilized to assess and monitor the progress of rehabilitation; nonetheless, they often present inherent limitations. In contrast, advancements in three-dimensional (3D) motion capture technology furnish detailed kinematic data, thereby augmenting the capacity to objectively quantify and elucidate movement deficits with heightened precision. This case highlights the critical significance of employing kinematic analysis with Xsens as an outcome measure to elucidate the intricacies of UCS, thereby offering invaluable insights for therapeutic interventions in similar clinical scenarios and providing objective insights into movement biomechanics, muscular function, and functional limitations. Leveraging this information, clinicians can skillfully tailor treatment modalities to address underlying musculoskeletal imbalances, ultimately optimizing patient outcomes. In this case study, we examine the kinematic analysis of a 48-year-old office worker experiencing persistent headaches, restricted range of motion, and neck and shoulder pain over a four-month period. Despite prior interventions, symptomatology deteriorated, prompting consultation with a neurophysiotherapist. The evaluation revealed localized pain in the right shoulder, upper back, and neck, characterized by gradual onset and dull ache, exacerbated by activity and alleviated by rest and medication, without diurnal fluctuations. Physical examination delineated UCS features. Following the implementation of a four-week physiotherapy rehabilitation protocol, initial assessments utilizing Xsens gait motion analysis were undertaken. Subsequent to the rehabilitation program, significant improvements were noted across various parameters. These encompassed augmented range of motion, heightened muscular strength, and enhanced flexibility. Additionally, discernible enhancements were observed in posture and gait, characterized by the restoration of normal cervical spine curvature and an expanded range of motion.

## Introduction

Upper cross syndrome (UCS) is a pathological condition characterized by heightened rigidity and diminished strength in the musculature of the neck, shoulders, and upper back, particularly in the region where they intersect the dorsal and ventral aspects of the body. While precise diagnostic criteria for UCS are not universally established, it is commonly identified by aberrant activation and movement patterns exhibited by the muscles governing the head, neck, shoulders, and back. Muscles such as the suboccipitalis, sternocleidomastoid, levator scapulae, pectoralis major and minor, scalene, and upper trapezius manifest tightness or shortening in individuals with UCS. Conversely, the deep neck flexors, serratus anterior, rhomboids, middle trapezius, and lower trapezius exhibit weakness, elongation, and restriction [1]. The presence of opposing muscle imbalances in UCS precipitates malalignments in the upper limbs and dysfunction in the atlantooccipital, cervicothoracic, and glenohumeral joints, subsequently contributing to postural irregularities [2]. Additionally, these muscular imbalances give rise to a spectrum of physical symptoms, including but not limited to headaches, neck discomfort, chest pain, upper back pain, tingling sensations in the upper arms, and constraints in the range of motion in the neck or shoulders [3,4].

An ideal posture is required for maximum functional performance in daily tasks. The uncomfortable positions continuously place strain on the joints, and when exposed to persistent pressure, degenerative changes in joints proceed quickly, leading to muscle imbalances and discomfort [5]. In simpler terms, this particular body posture results in the development of certain postural patterns, including forward shoulders, thoracic kyphosis, forward head, and a reduction in the natural curve of the neck (cervical lordosis). These deviations from the norm contribute to a collective alteration in the upper quarter of the body [6]. These aforementioned alterations have the potential to exert stress on the muscular attachments of the shoulder and scapula, consequently yielding a rounded appearance of the shoulders [7]. Kinematic analysis stands as a potent method for the objective evaluation of upper extremity movements within a three-dimensional (3D) space. Kinematic analysis plays a pivotal role in identifying specific muscle imbalances and aberrant movement patterns implicated in UCS, thereby guiding the development of targeted interventions aimed at rectifying these underlying issues. Moreover, it facilitates the ongoing monitoring of treatment efficacy and enables the optimization of therapeutic outcomes, ensuring a comprehensive approach to managing UCS and promoting patient well-being [8].

Maintaining a faulty posture for an extended period is the greatest risk factor for developing UCS. Many daily activities that cause poor posture are work-related. Postural abnormalities might become worse if you labor continuously or repetitively for lengthy periods. Patients with poor posture, for instance, are more likely than those with good posture to get musculoskeletal injuries after handling or moving large things [9]. The distinctive attributes of Xsens motion capture systems, encompassing portability, wireless connectivity, real-time feedback, high accuracy, and integration with biomechanical models, render it an invaluable instrument for kinematic analysis across diverse domains. Its utility spans from clinical rehabilitation to sports performance optimization and biomechanical inquiry, offering comprehensive insights into movement dynamics. Through its capabilities, Xsens enables tailored interventions aimed at enhancing outcomes, including those pertaining to UCS. Physiotherapy plays a vital role in treating UCS. It seeks to address postural imbalances, strengthen weak muscles, reduce discomfort, increase range of motion, and provide patients with the information and resources they need to maintain good posture and stop the illness from returning [10]. The present study identified the condition of UCS through kinematic analysis using Xsens and concentrated on neck, trunk, lumbopelvic complex, and arm swing components. Xsens represents a valuable tool in the assessment and management, offering a multifaceted approach to kinematic analysis that holds great potential for enhancing clinical decision-making and facilitates tailored interventions and optimization of patient outcomes. It focused on the results of the mechanism of gait in UCS.

## Case presentation

A 48-year-old office worker complained of constant headaches, limited range of motion, and persistent neck and shoulder pain for the last four months. The patient was alright one year back and arrived with a history of neck and shoulder discomfort that had been worsening during the previous year for which he went to a private hospital where medications were given for pain relief and he was sent back home. The patient frequently complained of headaches, especially following extended periods of desk work. The majority of his everyday tasks entailed him spending a lot of time in front of a computer. Pain has subsided for the time being but again reoccurs, and he was unable to extend his arm over 90° or perform his daily tasks correctly, so he came to the neurophysiotherapy department of the Acharya Vinoba Bhave Rural Hospital in Sawangi where assessments and investigations were conducted. On the visual analogue scale, the score was 5.8/10 on shoulder abduction movement and 1/10 on rest. The patient reported experiencing pain localized to the anterior aspect of the right shoulder, upper back, and neck. The pain manifested gradually, presenting as a dull ache. It was exacerbated by physical activity and alleviated by rest and medications. There were no diurnal variations. The physical examination indicated symptoms of UCS, including forward head position, rounded shoulders, upper trapezius, and levator scapulae muscle discomfort.

Clinical findings

Following the informed verbal consent, the patient underwent a thorough examination. The examination was conducted with the patient in a sitting position, during which no noteworthy abnormalities were observed in the vital signs. Upon local examination, the patient exhibited grade II tenderness on the anterior aspect of the right shoulder. A comprehensive physical assessment was performed, encompassing posture evaluation using a plumb line, revealing a posture characterized by rounded shoulders and a forward head position. Manual muscle testing (MMT) results are detailed in Table 1. Furthermore, the range of motion assessment is outlined in Table 2.

**Table 1:** MMT of the right upper limb and cervical muscles pre- and post-physiotherapy rehabilitation MMT: Manual muscle testing; 2+: elimination of gravitational forces or slight resistance, covering less than half the range against gravity; 3: complete range of motion against gravity; 3+: full range of motion against gravity with slight resistance; 4: full range of motion against gravity with moderate resistance; 4+: full range of motion against gravity with moderate to strong resistance

Manual Muscle Testing	Measurement	Pretreatment	Posttreatment
Shoulder	Flexors	3+/5	4+/5
	Abductors	3+/5	4/5
	Extensors	3+/5	4/5
Scapula	Elevators	3/5	4/5
Cervical	Flexors right side	2+/5	3+/5
	Flexors left side	3+/5	4/5
	Extensors right side	2+/5	3+/5
	Extensors left side	3+/5	4/5
	Rotators right side	2+/5	4/5
	Rotators left side	3+/5	4/5

**Table 2:** ROM of the right upper limb and cervical joints pre- and post-physiotherapy rehabilitation ROM: Range of motion; AROM: active range of motion; PROM: passive range of motion

Range of Motion	Measurement	AROM		PROM	
		Pretreatment	Posttreatment	Pretreatment	Posttreatment
Shoulder	Flexion	0˚-105˚	0˚-125˚	0˚-112˚	0˚-130˚
	Abduction	0˚-95˚	0˚-110˚	0˚-100˚	0˚-120˚
	Extension	20˚-0˚	25˚-0˚	22˚-0˚	28˚-0˚
Cervical	Flexion	0˚-20˚	0˚-30˚	0˚-25˚	0˚-38˚
	Extension	0˚-35˚	0˚-48˚	0˚-42˚	0˚-52˚
	Lateral flexion right side	0˚-15˚	0˚-25˚	0˚-20˚	0˚-30˚
	Lateral flexion left side	0˚-25˚	0˚-30˚	0˚-32˚	0˚-35˚
	Rotation right side	0˚-30˚	0˚-30˚	0˚-35˚	0˚-35˚
	Rotation left side	0˚-45˚	0˚-45˚	0˚-54˚	0˚-55˚

Therapeutic intervention

**Figure 1:**
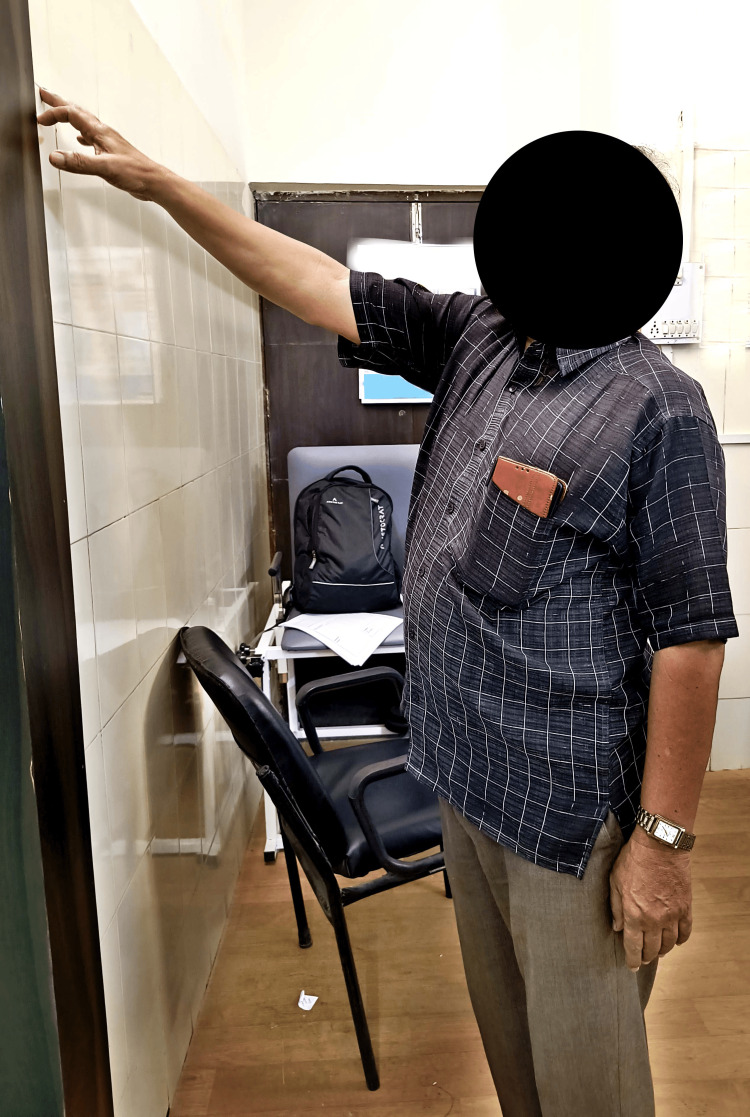
Patient performing finger ladder exercise

**Figure 2:**
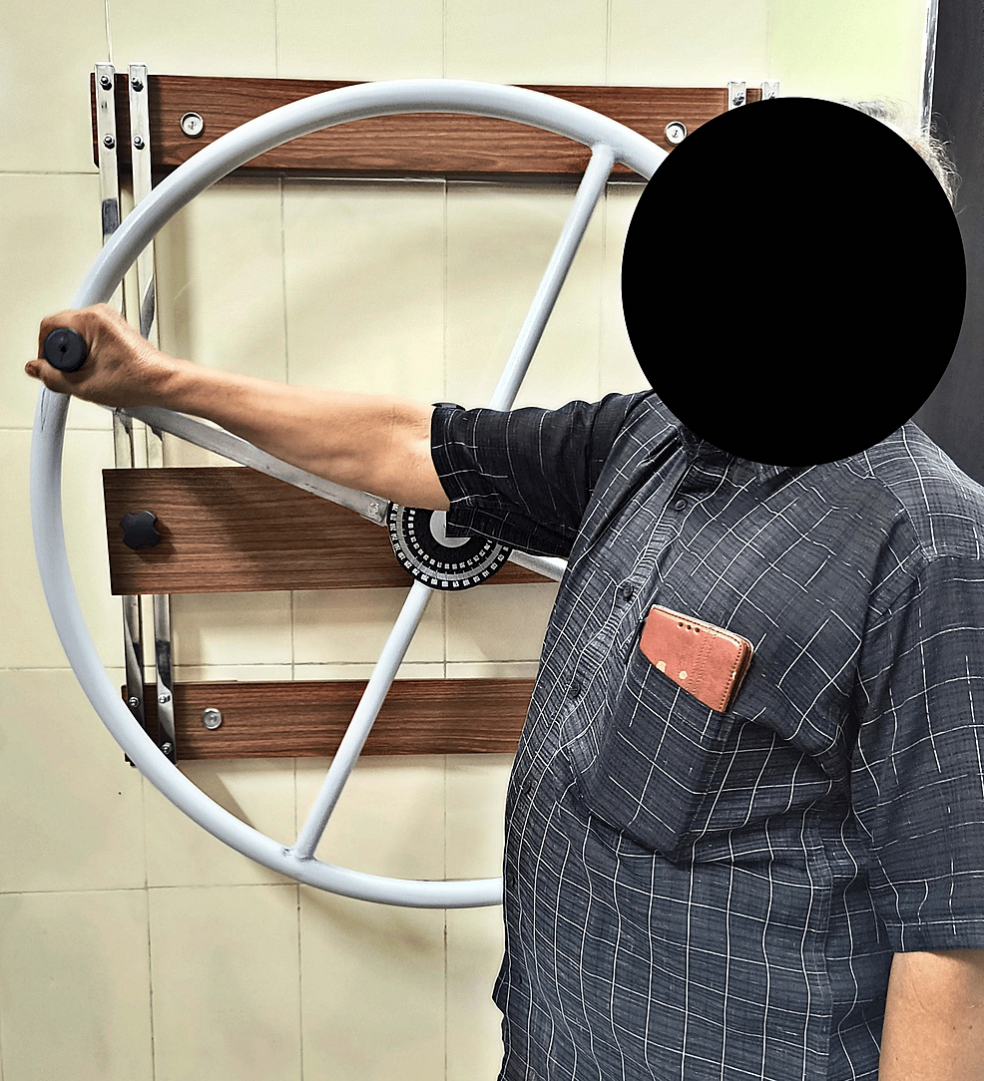
Patient performing shoulder wheel exercise

A personalized therapeutic plan is given to improve the range and functional activities of patient given in Table 3. Figure 1 and Figure 2 show the patient performing finger ladder exercise and shoulder wheel exercise, respectively, to increase the range of motion.

**Table 3:** Therapeutic intervention for four weeks IFT: Interferential therapy; ROM: range of motion

Goals	Interventions Weeks 0-4
Pain relief	Hydro collateral pack before treatment (15 minutes) IFT (4 pole vector) for 10 minutes
Improve ROM	Finger ladder, shoulder wheel, mobilization (glenohumeral posterior glide to elevate flexion, glenohumeral caudal glide to elevate abduction, and glenohumeral anterior glide to elevate extension)
Improve flexibility	Stretching for the pectoralis major muscle with 10 seconds hold (5 repetitions x 1 set)
Improve strength	Muscle energy technique on the sternocleidomastoid and upper trapezius muscle (5 repetitions using 20% of maximal isometric contraction for 20 seconds). Scapular sets with red Thera band (10 repetitions x 1 set)
Functional activities	Functional reach outs (10 repetitions on each side x 1 set)
Ergonomic modifications	Education regarding the breaks for stretching or walking, seat-pan angle, and desktop height at eye level was given and modifications were planned

Follow-up outcomes

**Figure 3:**
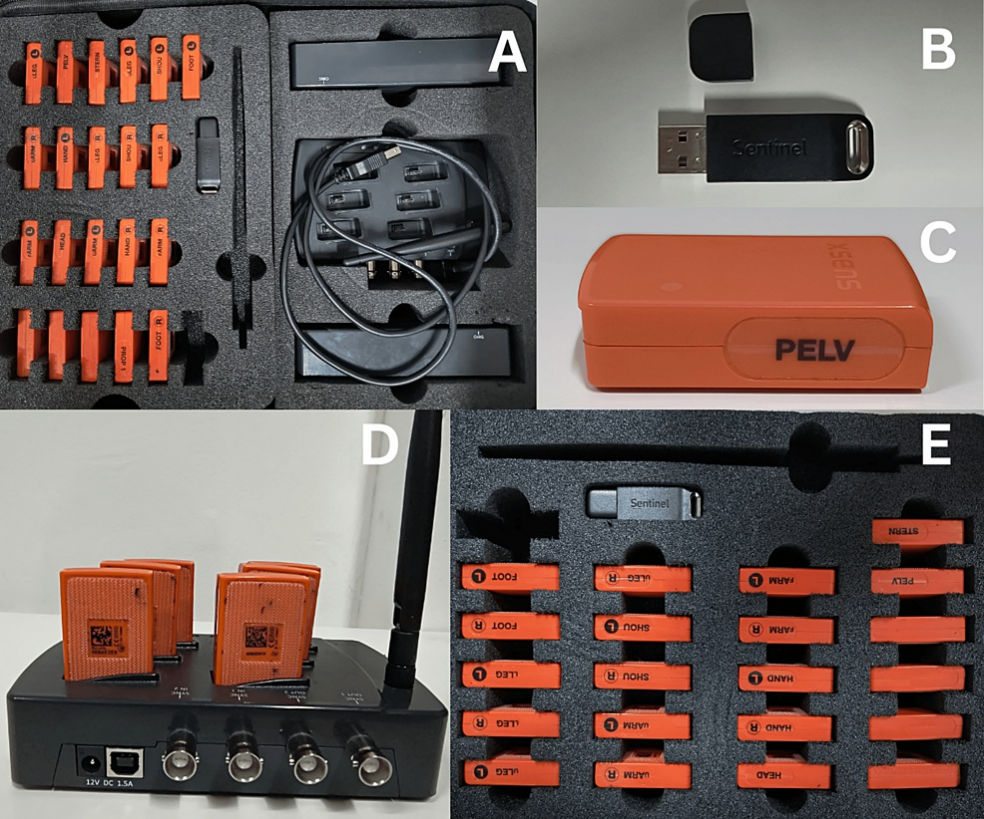
(A) Xsens kit, (B) dongle, (C) sensor, (D) charging portal, (E) 21 sensors

**Figure 4:**
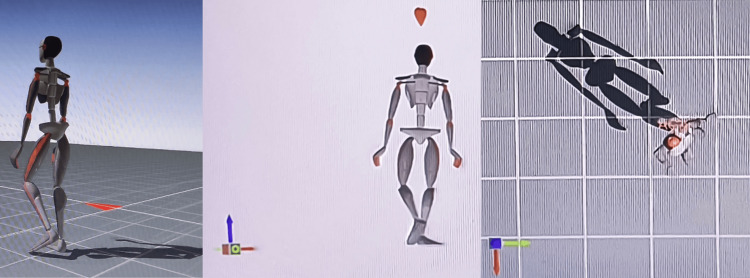
Avatar movements in three views

Follow-up and outcomes after the treatment using gait motion analysis are shown in Table 4. Kinematic analysis via Xsens involves algorithmically derived projections, presenting estimations rather than precise values. This methodology relies upon a comprehensive 3D postural assessment. Video analysis of patient motions is executed along the three spatial axes (x, y, and z). Xsens motion capture systems play a pivotal role in digitizing human movement. This sophisticated software meticulously records, monitors, and evaluates movement patterns. The hardware component, MVN Link, is accessed through a dongle. Xsens employs advanced inertial sensor technology for precise orientation, attitude, and positioning data. Each setup comprises 21 sensors, with 17 affixed to the body and four additional sensors. The patient's bodily movements are seamlessly integrated into avatars in real time, thereby immersing their entire body within virtual environments. Utilizing wearable motion capture systems, MVN Analyze software, and automated cloud-based reports, practitioners can attain precise kinematic data and assess subjects across various environments, including nonlaboratory settings. The Xsens hardware component is depicted in Figure 3, while avatar movements are illustrated in Figure 4.

**Table 4:** Kinematic analysis of normal person and UCS patient before and after the treatment UCS: Upper cross syndrome

Variables	Normal Person	Baseline	Posttreatment
	Z- axis	Z- axis	Z- axis
Neck segment position	1.39	1.29	1.39
Center of mass	0.91	0.85	0.91

## Discussion

In the context of cervical segment positioning, individuals diagnosed with UCS exhibit compromised mobility, resulting in a pronounced limitation of movement along a specific axis relative to others. Nevertheless, the restoration of movement should ideally manifest proportionately across all three axes x, y, and z. Following the therapeutic intervention, it becomes evident that the regained mobility is distributed uniformly across these axes. Furthermore, significant alterations in the center of mass predominantly manifest along the z-axis among affected individuals, with values notably lower than those observed in healthy counterparts. Posttreatment for UCS, a marked enhancement across all three axes is observed, reflecting an overall improvement in functional mobility and balance.

Gait is an essential clinical evaluation tool, since alterations in gait may be indicative of alterations in overall health. Recognizing the significance of gait as a barometer of physiological well-being, we employed Xsens gait motion analysis to scrutinize and interpret gait patterns [11]. Within the population exhibiting upper cross syndrome, the implementation of the muscle energy technique has demonstrated heightened efficacy in alleviating pain, improving range of motion, and optimizing functional outcomes in comparison to conventional stretching exercises [12]. Researchers delve into the examination of locomotion patterns, balance, and joint kinematics in individuals afflicted with movement disorders, which can stem from a spectrum of underlying causes, including musculoskeletal, neurological, or systemic dysfunctions. Physiotherapists play a pivotal role in addressing movement disorders by providing interventions aimed at reducing pain, enhancing joint mobility and muscle strength, improving postural stability, refining locomotion quality, and minimizing fall risk [13]. The implementation of exercise regimens endorsed by the National Academy of Sports Medicine, alongside strategic ergonomic interventions, holds promise as an effective rehabilitation protocol, particularly in mitigating forward head posture, protracted shoulder positioning, and thoracic kyphosis [14].

Xsens inertial sensors present a valuable tool for the objective assessment of patient movement and progress throughout the rehabilitation continuum. Through real-time motion data capture, clinicians can monitor enhancements in range of motion, joint stability, and functional movement patterns. This empirical data furnishes crucial insights into the efficacy of the rehabilitation regimen, enabling timely adjustments as warranted. A notable advantage of Xsens technology lies in its capacity to facilitate personalized rehabilitation protocols. By scrutinizing the patient's specific movement patterns and biomechanics, clinicians can tailor the rehabilitation regimen to address individual deficits and hurdles. This individualized approach is paramount for optimizing outcomes and mitigating the risk of complications or recurrent injury [15]. While Xsens inertial sensor technology presents numerous advantages for rehabilitation, it is important to acknowledge its accompanying challenges and limitations. Factors including cost, accessibility, and the requisite technical expertise for implementation may impede its widespread adoption. Moreover, the accuracy and reliability of motion capture data can be affected by variables such as sensor placement, calibration procedures, and environmental conditions. Clinicians must exercise vigilance regarding these limitations and judiciously integrate inertial sensor data with other clinical assessments to ensure informed decision-making in patient care [16].

Physiotherapists routinely assess rehabilitation outcomes through subjective means, utilizing visual observation, clinical judgment, and various tests and measures. Recent advancements in rehabilitation assessment involve the utilization of innovative technologies, including external sensors, smartphones, and wearable sensors. These technologies facilitate the objective evaluation of rehabilitation progress, with external sensors strategically positioned in the patient's environment, while smartphones and wearable sensors are directly affixed to the patient, enabling diverse methods of precise data collection [17]. The head postures (head tilt and craniovertebral angle), shoulder posture (sagittal shoulder angle), and trunk posture (trunk flexion angle) were recorded using a gait motion analysis Xsens device. The analysis revealed forward head posture, reduced arm swing on the right side, and decreased range of motion in the right shoulder joint before physiotherapy treatment. Posttreatment, the recordings exhibited a restoration of normal cervical spine curvature and an increase in the range of motion, indicating positive therapeutic outcomes.

## Conclusions

This case report underscores the invaluable role of kinematic analysis, particularly through the utilization of Xsens technology, in the assessment of UCS. Through the integration of sophisticated motion capture technology, this case report exemplifies the potential for precise and objective characterization of movement abnormalities associated with UCS. The utilization of Xsens offers valuable insights into gait dynamics, aiding in the identification of specific impairments and guiding tailored interventions for improved patient outcomes. However, it is imperative to acknowledge the inherent limitations of such technology, including cost considerations and technical expertise requirements. Nonetheless, the findings underscore the utility of kinematic analysis in enhancing our understanding of UCS and optimizing therapeutic strategies, heralding a paradigm shift toward personalized and evidence-based care in musculoskeletal rehabilitation. An individualized physical therapy intervention plan with ergonomical modification was created to treat muscular imbalances and postural problems, and using a diagnostic approach, a kinematic analysis via Xsens shows greater improvement in patients with UCS indicating improved range of motion with restoration of normal curvature of cervical spine which improved the quality of life.
